# Leprosy as Immune Reconstitution Inflammatory Syndrome in HIV-positive Persons

**DOI:** 10.3201/eid1309.070301

**Published:** 2007-09

**Authors:** Frank Martiniuk, Shaline D. Rao, Thomas H. Rea, Michael S. Glickman, Jerome Giovinazzo, William N. Rom, Aloys Cabrera, William R. Levis

**Affiliations:** * New York University School of Medicine, New York, New York, USA; † University of Southern California, Los Angeles, California, USA; ‡ Memorial Sloan Kettering Cancer Center, New York, New York, USA; § Amherst College, Amherst, Massachusetts, USA; 1 These authors contributed equally to this article.

**Keywords:** HAART, Mycobacterium leprae, subclinical, HIV, heat shock 65, *hsp65*, leprosy, RFLP, letter

**To the Editor:** More than 2 decades ago, when HIV was first detected, many investigators predicted the rise of leprosy secondary to opportunistic infection (*1*). Recently, the phenomenon of immune reconstitution inflammatory syndrome (IRIS), or leprosy reversal response, has received attention. IRIS often occurs secondary to initiating highly active antiretroviral therapy (HAART). The first indications of an interaction between HIV and *Mycobacterium leprae* occurred only recently, with the identification of IRIS after initiation of HAART in patients with HIV and previously undetected leprosy. A review by Pustianowski et al. discusses the paradox of HIV and leprosy with IRIS (*2*). In addition, Lawn et al. described the first case of IRIS after the onset of HAART in a patient who had tuberculoid leprosy that was never confirmed by molecular analysis (*3*).

Multiple reports (*4*–*7*) unmasked subclinical Hansen disease (*M. leprae* infection) occurring with HAART or spontaneously (*8*). In case reports by Lu et al. (*6*) and Sharma et al. (*7*), leprosy was associated with erythema nodosum leprosum. Pereira et al. discovered that patients known to have HIV and leprosy, when treated with HAART manifested a type 1 reversal reaction, acute leprosy inflammatory episode (*4*), or IRIS. We describe the first, to our knowledge, 2 cases in the United States of HIV and leprosy infections in which IRIS has occurred after HAART initiation and which has been confirmed by molecular analysis.

Three skin-biopsy samples, 2 from patient 1 and 1 from patient 2, were analyzed to confirm the presence of *M. leprae*. Patient 1 met the diagnostic criteria for leprosy according to biopsy result; patient 2’s case was compatible with such criteria. Each patient was treated for leprosy, and each responded favorably. The purpose of our case study was to confirm *M. leprae* DNA in skin samples. The skin specimens were paraffin-embedded slides. DNA was extracted by standard molecular biologic methods that used xylene. PCR amplified the *M. leprae* heat shock protein 65 gene (*hsp65*). After amplification, restriction fragment-length polymorphism (RFLP)–polyacrylamide gel electrophoresis (PAGE) was performed with *Hae*III (*6*).

Patient 1 was a 60-year-old Hispanic man who was first evaluated in Los Angeles, California, with skin lesions covering >50% of his body. He reported having erythematous scaly plaques that had been waxing and waning for several months. Several skin biopsy samples were taken, and an HIV test was conducted; results showed that he had lepromatous leprosy and was HIV positive. Biopsy specimens were both Fite stain positive for numerous acid-fast bacilli. Three months after HAART was initiated, repeat skin biopsy samples were taken from nodules that had recently developed on his right arm and torso. Histologic assessment showed Fite stain–positive granulomatous dermatitis with many foamy cells. He was treated for leprosy and is continuing HAART.

Patient 2 was a 37-year-old West African black man from Burkina Faso who was evaluated in New York for gram-negative bacteremia. He was admitted and treated for disseminated salmonellosis and was found to be HIV positive. His T-lymphocyte count was 7/μL. He was promptly prescribed HAART and responded well to treatment: his T-cell count rose to 112/μL during 5 months and is currently >700/μL. Within 2 years of HAART initiation, multiple anesthetic, hypopigmented skin macules that failed to resolve over 6 months developed. These macules developed further into nodules. Punch biopsy results were consistent with granulomatous dermatitis. Fite stain was negative for acid-fast bacilli, but leprosy was diagnosed on the basis of anesthesia localized to his skin lesions. When the biopsy samples were taken, the patient was receiving dapsone in addition to HAART. Rifampin treatment was started subsequent to biopsies.

PCR amplification for *M. leprae* *hsp65* was positive for all 3 samples. Thus, mycobacterial DNA was present in both patients. The RFLP analysis results are shown in the Figure. The *hsp65* RFLP pattern for patient 1 was identical to those described by Martiniuk et al. (*9*) and for the wild-type pattern for patient 2, as shown by Lu et al. (*6*), thus demonstrating the presence of *M. leprae* DNA in these samples.

**Figure:**
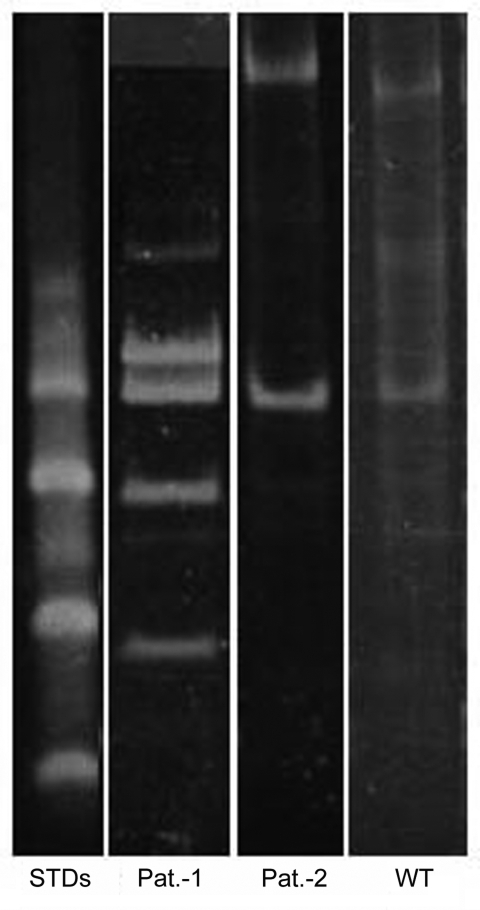
Polyacrylamide gel electrophoresis–restriction fragment length polymorphism of PCR amplicons digested with *Hae*III with standards. STDs, sexually transmitted diseases; Pat., patient; WT, wild type.

Previous studies have highlighted the low rate of HIV and leprosy co-infection. For example in Ethiopia, Frommel et al. noted that, before HAART was available in resource-poor settings, increased HIV seropositivity did not alter the natural course of leprosy nor increase the number of patients with *M. leprae* (*10*). Nevertheless, positive reports of IRIS and leprosy after initiation of HAART have been reported from other nations (*3*–*5*). If this syndrome can be detected even in the mildly leprosy–endemic United States (*8*), an increase in similar cases in areas where HIV and leprosy occur in higher frequency can be anticipated.
